# Incidence, Risk Factors, and Outcomes of Perioperative Atrial Fibrillation following Noncardiothoracic Surgery: A Systematic Review and Meta-Regression Analysis of Observational Studies

**DOI:** 10.1155/2021/5527199

**Published:** 2021-04-28

**Authors:** Yamini Subramani, Omar El Tohamy, Daniil Jalali, Mahesh Nagappa, Homer Yang, Ashraf Fayad

**Affiliations:** ^1^Department of Anesthesia and Perioperative Medicine, London Health Sciences Centre and St. Joseph Health Care, Western University, Schulich School of Medicine and Dentistry, London, Ontario, Canada; ^2^Department of Medical Sciences, Western University, London, Ontario, Canada

## Abstract

**Background:**

Atrial fibrillation (AF) occurs in 16–30% of patients after cardiac and thoracic surgery and can lead to serious complications like hypoperfusion of vital organs, pulmonary edema, and myocardial infarction. The evidence on risk factors and complications associated with perioperative AF after noncardiothoracic surgery is limited.

**Methods:**

The primary objective was to determine demographic and clinical risk factors for new-onset atrial fibrillation associated with noncardiothoracic surgery. A secondary aim was to identify the incidence and odds of perioperative complications associated with the new-onset atrial fibrillation. A systematic search within multiple databases was conducted for studies that explicitly reported on new-onset atrial fibrillation after noncardiothoracic surgery. We reported data on demographics, comorbidities, and perioperative complications as mean difference (MD) or odds ratios (OR) and corresponding 95% confidence interval (CI) using random effects models. A two-sided *P* value of less than 0.05 was considered significant. We performed meta-regression and sensitivity analysis of various subgroups to confirm the inference of our findings.

**Results:**

Eleven studies reporting on 121,517 patients were included, of whom 2,944 developed perioperative AF (incidence rate: 3.7%; 95% CI: 2.3%––6.2%). Advanced age (AF group versus control group: 69.36 ± 10.5 versus 64.37 ± 9.53 years; MD: 4.06; 95% CI: 1.67––6.44; *P*=0.0009), male gender (52.85% versus 43.59%; OR: 1.08; 95% CI: 0.54 to 1.62; *I*^2^: 84%; *P* < 0.0001), preoperative hypertension (60.42% versus 56.51%; OR: 1.15; 95% CI: 1.08 to 1.23; *I*^2^: 0%; *P* < 0.00001), diabetes mellitus (22.6% versus 23.04%; OR: 0.97; 95% CI: 0.89 to 1.05; *I*^2^: 0; *P* < 0.00001), and cardiac disease (30.64% versus 8.49%; OR: 2.3; 95% CI: 0.28 to 4.31; *I*^2^: 93%; *P*=0.03) were found to be significant predictors for perioperative AF. The AF group was at increased odds of developing postoperative cardiac complications (34.1% versus 5%; OR: 5.44; 95% CI: 0.49 to 10.39; *I*^2^: 82%; *P*=0.03), postoperative stroke (0.5% versus 0.1%; OR: 3; 95% CI: 0.65 to 5.35; *I*^2^: 0%; *P*=0.01), and mortality (7.40% versus 1.92%; OR: 3.58; 95% CI: 0.14 to 7.02; *I*^2^: 0%; *P*=0.04). Study quality assessment by meta-regression and sensitivity analysis of the various subgroups did not affect the final inference of the results.

**Conclusion:**

We identified advanced age, male gender, preoperative hypertension, diabetes mellitus, and cardiac disease as important risk factors for perioperative atrial fibrillation. The atrial fibrillation group was at increased odds for postoperative cardiac complications, stroke, and higher mortality, emphasizing the need for risk stratification and close monitoring.

## 1. Introduction

Atrial fibrillation (AF) occurs in 16–30% of patients after cardiac and thoracic surgery [1–4], secondary to direct mechanical irritation of the myocardium or pericardium. Perioperative AF is associated with an increased risk of in-hospital morbidity and mortality [5]. Atrial arrhythmias are the most frequent rhythm disturbances in the postoperative period [6], and ventricular arrhythmias and brady arrhythmias are less frequent. The pathophysiology of AF associated with noncardiothoracic surgery is poorly understood but is thought to be due to inflammatory postoperative response triggering a disorganized electrical activity within atrial myocytes [7]. In addition, surgery and anesthesia are associated with a stress-induced increased sympathetic activity, thereby predisposing the patient to arrhythmias [8, 9]. There are several studies in the literature which largely focus on perioperative AF following cardiothoracic surgery [3, 4, 10, 11]. But the evidence concerning perioperative AF following noncardiothoracic surgery is limited. An increase in the number of ageing surgical populations over the last decade has increased the overall prevalence of postoperative AF [12–14]. Hence, it is important to identify the risk factors and outcomes associated with perioperative AF to characterize those patients at risk of postoperative complications. The primary objective of this systematic review and meta-analysis (SRMA) is to identify the risk factors associated with perioperative AF during noncardiothoracic surgery. The secondary objective is to identify any perioperative complications associated with perioperative AF.

## 2. Methods

This SRMA was conducted with a predesigned protocol (Supplementary File S1), which is registered at PROSPERO (CRD42019131060). This meta-analysis is reported as per the Preferred Items for Systematic Reviews and Meta-Analyses (PRISMA) guidelines [15].

### 2.1. Study Selection

We included studies which reported on perioperative AF in adult patients (>18 years), along with a control group, after noncardiothoracic surgery. Perioperative AF is defined as that identified in the intraoperative/postoperative period, as a single occurrence on electrocardiogram (ECG) or a series of recordings on a 24-h ECG, with an onset within 30 days of the surgery. We did not have a fixed ECG definition for the AF, which could be symptomatic or asymptomatic and paroxysmal or persistent. We excluded case series, case reports, and any study without explicit and exclusive reporting of perioperative AF. We also excluded studies that were conducted in the cardiac and thoracic surgery setting and those studies reporting on patients with documented AF that occurred before the surgical procedures as our aim was to identify new-onset perioperative AF associated with noncardiothoracic surgeries. Abstracts and conference publications were excluded as they were not deemed to have undergone an adequate peer review process, and studies that were not published in English language were also excluded due to resource limitations.

### 2.2. Search Strategy

Based on predefined search criteria, an expert librarian systematically searched the following electronic databases: PubMed, Medline, Embase, Web of Science, and Cochrane databases, using the following terms and combinations of keywords, per the National Center for Biotechnology Information Medical Subject Headings (NCBI MeSH): “after surgery” or “following surgery” or “post-surgery” or “post-transplant” or “perioperative” or “periprocedural” or “intraoperative” or “intraprocedural” or “postoperative” or “postprocedural” or “perioperative period” or “arrhythmia” or “bradycardia” or “tachycardia” or “atrial flutter” or “atrial fibrillation” or “afib” or “dysrhythmia” or “tachyarrhythmia” or “bradyarrhythmia.” The search was run on February 2019 and updated February 2020. The search strategy is attached as a supplementary file (S2). Three authors (OE, DJ, and YS) independently scrutinized the list of titles and abstracts, to sort out the articles to be included in the SRMA. After this, full texts of the positively screened articles were retrieved and independently assessed by two reviewers (DJ and OE) for the inclusion criteria. In case of any conflict, the senior author (AF) was consulted. Additionally, the reference lists of the included studies were hand searched for any relevant articles to be included. A modified Newcastle–Ottawa scale [16] and Quality in Prognostic Studies (QUIPS) tool [17] were used to assess the quality of the included studies.

### 2.3. Data Extraction

A data collection form was designed and the following data were extracted: study characteristics, including name of the author, publication year, study type, and participant number; preoperative data including age, sex, preoperative medications, cardiovascular, respiratory and general medical comorbidities, like diabetes mellitus, and hypertension; intraoperative data including type of surgery, duration, blood loss, and intraoperative cardiopulmonary complications; and postoperative data including postoperative complications like cardiopulmonary events, postoperative sepsis, stroke, and mortality. The definition and timing of the perioperative AF were recorded. The abovementioned were collected using a standardized data collection proforma. The authors YS and AF confirmed the accuracy and completeness of all the data.

### 2.4. Outcome Definition

The primary outcome was the demographic and clinical risk factors for new-onset perioperative AF with noncardiothoracic surgery. Secondary outcomes were the incidence and perioperative complications associated with the perioperative AF.

### 2.5. Quantitative Data Synthesis

Data on demographics, comorbidities, and perioperative complications were analysed by extracting and pooling odds ratios and mean differences using an inverse variance statistical method that incorporates a measure of the extent of heterogeneity into study weights, following DerSimonian and Laird's method. Continuous data were reported as mean difference (MD). Dichotomous data were reported as odds ratio (OR) and 95% confidence interval (CI). A two-sided *P* value of less than 0.05 was considered significant. Unless otherwise stated, we pooled unadjusted odds ratios. A pooled incidence of AF was estimated employing an epidemiological random effects model, using an inverse variance statistic that incorporates a measure of the extent of heterogeneity into study weights, following DerSimonian and Laird's method. Egger's test, Begg's test, fail-safe *N*-test, and inspection of the funnel plot were done to assess publication bias.

Each analysis was assessed for statistical heterogeneity using the *I* [2] statistic [18] and chi-square test. *I* [2] values >50% and *P* < 0.05 for the chi-square test indicate significant heterogeneity. A random effects model was used for all analyses to account for the between-study heterogeneity. Heterogeneity was further explored with an influence analysis for significant risk factors and outcomes.

An influence analysis was performed by excluding each study in the analysis for the significant risk factors and outcomes, and the pooled estimates were recalculated. If the quality or eligibility of any of the studies was in doubt, the analysis was performed by both including and excluding these studies to check the sensitivity of the pooled estimates. Study quality assessment was conducted (as categorical variable) by meta-regression and sensitivity analysis of various subgroups based on the study type (retrospective versus prospective), quality of study (good versus poor-moderate), clearly defined outcomes (yes versus no), and sample size >1000 (yes versus no), and type of surgery (Transplant versus nontransplant procedure). The analysis was conducted using the Review Manager software (RevMan, V.5.3) and Comprehensive Meta-Analysis (CMA) software.

## 3. Results

**Figure 1:**
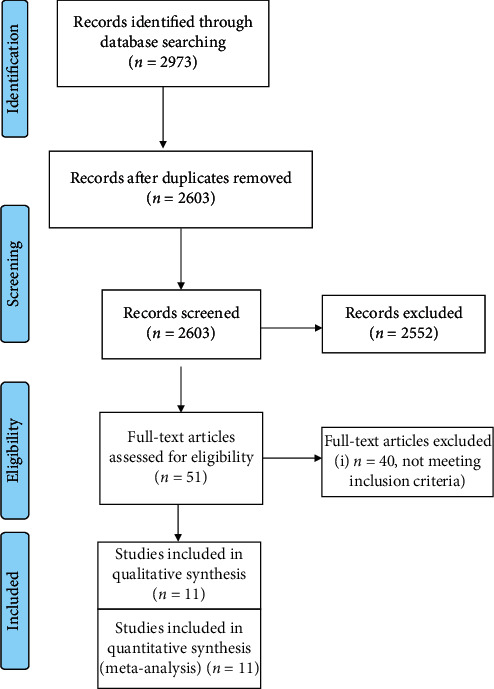
PRISMA flow diagram showing the articles screened for eligibility as per the inclusion criteria to be included in the systematic review and meta-analyses.

Our initial search identified 2,973 studies, and after removing duplicates, 2,603 were screened, by titles and abstracts, to yield 51 studies for full-text eligibility review. Forty studies were excluded as they did not meet the eligibility criteria. Finally, 11 studies met the inclusion criteria and were included in this SRMA (Figure 1) [19–29]. These studies were analysed with respect to risk factors and outcomes associated with the perioperative AF. The definition of perioperative AF varied across the studies (Supplementary File S3). The 11 included studies reported on 121,517 patients undergoing noncardiothoracic surgery, of whom 2,944 developed perioperative AF. The pooled incidence of perioperative AF was 3.7% (95% CI: 2.3%––6.2%). Supplementary File S4 summarizes the systematic review of potential risk factors categorized as demographics, medical comorbidities, and postoperative complications. The quality of the included studies assessed using the modified Newcastle–Ottawa scale [16] yielded scores between 7 and 9, indicating a low risk of bias (Supplementary File S5). Supplementary File S6 shows a quality analysis using the QUIPS tool. There was a moderate to high risk of bias in confounding factor measurement as these factors were often poorly defined and measured. There was a low to moderate risk of bias in outcome measurement, as the methods to identify perioperative AF were well described in the studies. There was a low to moderate risk of bias in statistical analysis where multivariate analyses were conducted.

### 3.1. Demographics

**Figure 2:**
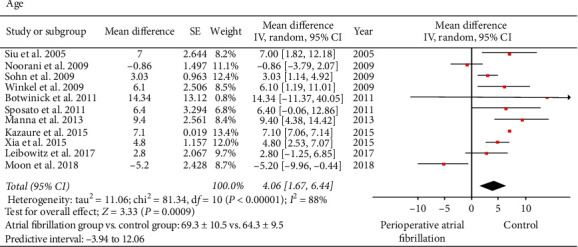
Forest plot evaluating age as a risk factor for perioperative atrial fibrillation in patients undergoing noncardiothoracic surgery. The mean difference of each included study is plotted. A pooled estimate of overall mean difference (diamonds) and 95% confidence intervals (width of diamonds) summarizes the effect size using the random effects model. CI = confidence interval; IV = inverse variance; MD = mean difference; *I*^2^: heterogeneity; *P* < 0.05 is significant.

Regarding age, eleven studies involving 121,517 patients reported on age: 2,944 patients in the AF group and 118,573 patients in the control group [19–29]. The average mean age in the AF group was 69.36 ± 10.5 years compared to 64.37 ± 9.53 years in the control group. Age is a significant predictor of perioperative AF (MD: 4.06; 95% CI: 1.67 to 6.44; *I*^2^: 88%; *P*=0.0009) (Figure 2). The impact of the Kazaure et al. study [21], explored with influence analysis, showed that MD slightly decreased and heterogeneity decreased, without changing the final inference of the study (MD: 3.54; 95% CI: 1.16 to 5.92; *I*^2^: 72%; *P*=0.004). Egger's regression test (*P*=0.0001) and Begg's rank test (*P*=0.029) suggested the possibility of a publication bias; however, inspection of the funnel plot and fail-safe *N* test (*n* = 4433) did not indicate any publication bias.

**Figure 3:**
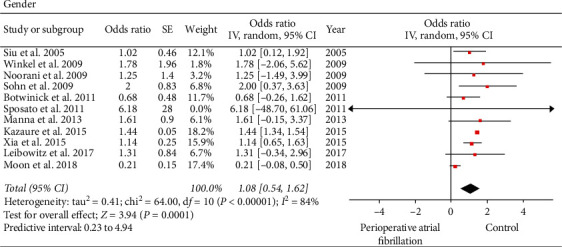
Forest plot evaluating male gender as a risk factor for perioperative atrial fibrillation in patients undergoing noncardiothoracic surgery. The odds ratio of each included study is plotted. A pooled estimate of overall odds ratio (diamonds) and 95% confidence intervals (width of diamonds) summarizes the effect size using the random effects model. CI = confidence interval; M–H = Mantel–Haenszel; OR = odds ratio; *I*^2^: heterogeneity; *P* < 0.05 is significant.

Regarding gender, eleven of the included studies consisting of 121,517 patients reported on gender (perioperative AF versus control: 2944 versus 118,573) [19–29]. There was a higher incidence of perioperative AF in males versus females (perioperative AF versus control: 1556 versus 51692: 52.85% versus 43.59%; OR: 1.08; 95% CI: 0.54 to 1.62; *I*^2^: 84%; *P* < 0.0001) (Figure 3). When influence analysis was performed to investigate the impact of the study by Kazaure et al. [21], the OR slightly decreased and heterogeneity decreased without impacting the final inference of the results (OR: 0.92; 95% CI: 0.45 to 1.40; *I*^2^: 49%; *P*=0.0001). Egger's test (*P*=0.322) and Begg's test (*P*=0.876) did not show any publication bias.

Regarding body mass index (BMI), there was no significant difference in BMI between the perioperative AF and control group. The mean BMI was 26.67 ± 5.52 for the perioperative AF group compared to 24.97 ± 3.98 among the control group (MD: 0.18; 95% CI: −1.14 to 1.50; *I*^2^: 84; *P*=0.79) [19, 25, 27, 29].

### 3.2. Medical Comorbidities

#### 3.2.1. Hypertension

**Figure 4:**
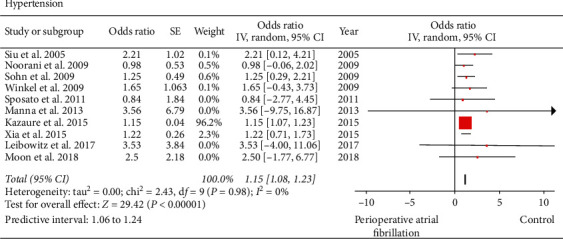
Forest plot evaluating hypertension as a risk factor for perioperative atrial fibrillation in patients undergoing noncardiothoracic surgery. The odds ratio of each included study is plotted. A pooled estimate of overall odds ratio (diamonds) and 95% confidence intervals (width of diamonds) summarizes the effect size using the random effects model. CI = confidence interval; M–H = Mantel–Haenszel; OR = odds ratio; *I*^2^: heterogeneity; *P* < 0.05 is significant.

Ten studies consisting of 121,268 patients reported on hypertension [19, 21–29]. In AF group, out of 2931 patients, 1771 patients had hypertension, while in the control group, out of 118,337 patients, 66,876 patients had hypertension. We found that hypertension (perioperative AF versus control: 60.42% versus 56.51%; OR: 1.15; 95% CI: 1.08 to 1.23; *I*^2^: 0%; *P* < 0.00001) was significantly associated with perioperative AF (Figure 4). When influence analysis was performed to investigate the impact of the study by Kazaure et al. [21], the OR slightly increased and heterogeneity decreased without impacting the final inference of the results (OR: 1.26; 95% CI: 0.87 to 1.65; *I*^2^: 0%; *P* < 0.00001). Egger's regression test (*P*=0.02684) suggested the possibility of a publication bias; however, Begg's rank test (*P*=0.37109), inspection of the funnel plot, and fail-safe *N* test (*n* = 27) did not indicate any publication bias.

#### 3.2.2. Diabetes Mellitus

**Figure 5:**
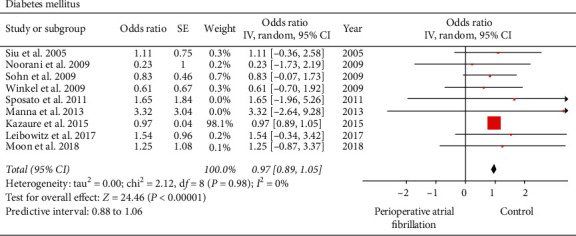
Forest plot evaluating diabetes mellitus as a risk factor for perioperative atrial fibrillation in patients undergoing noncardiothoracic surgery. The odds ratio of each included study is plotted. A pooled estimate of overall odds ratio (diamonds) and 95% confidence intervals (width of diamonds) summarizes the effect size using the random effects model. CI = confidence interval; M–H = Mantel–Haenszel; OR = odds ratio; *I*^2^: heterogeneity; *P* < 0.05 is significant.

Nine of the included studies reported on diabetes [19, 21–24, 26–29]. In AF group, out of 2847 patients, 643 patients had diabetes mellitus, while in the control group, out of 117,052 patients, 26,974 patients had diabetes mellitus. Diabetes mellitus was a significant predictor of perioperative AF (perioperative AF versus control: 22.6% versus 23.04%; OR: 0.97; 95% CI: 0.89 to 1.05; *I*^2^: 0; *P* < 0.00001) (Figure 5). When influence analysis was performed to investigate the impact of the study by Kazaure et al. [21], the OR slightly increased without impacting the final inference of the results (OR: 0.92; 95% CI: 0.35 to 1.48; *I*^2^: 0%; *P*=0.001). Begg's test (*P*=0.91697) and Egger's regression test (*P*=0.46851) did not indicate presence of publication bias.

#### 3.2.3. Cardiac Disease

**Figure 6:**
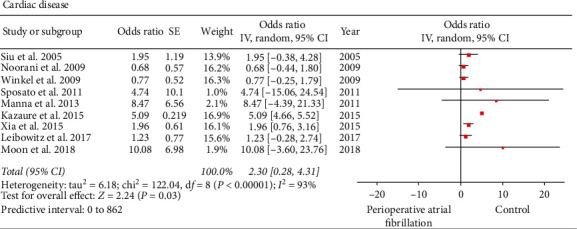
Forest plot evaluating cardiac disease as a risk factor for perioperative atrial fibrillation in patients undergoing noncardiothoracic surgery. The odds ratio of each included study is plotted. A pooled estimate of overall odds ratio (diamonds) and 95% confidence intervals (width of diamonds) summarizes the effect size using the random effects model. CI = confidence interval; M–H = Mantel–Haenszel; OR = odds ratio; *I*^2^: heterogeneity; *P* < 0.05 is significant.

Nine of the included studies with 113,512 reported on the incidence of preoperative cardiac disease (perioperative AF versus control: 2901 versus 110,611) [19, 21–26, 28, 30]. The presence of a cardiac disease was higher in the perioperative AF group versus control group (perioperative AF versus control: 889 versus 9399; 30.64% versus 8.49%; OR: 2.3; 95% CI: 0.28 to 4.31; *I*^2^: 93%; *P*=0.03) (Figure 6). Preoperative cardiac disease included ischemic heart disease, valvular heart disease, and congestive heart failure. Supplementary File S4 provides individual study data on the preoperative cardiac comorbidities in both the AF and control groups. When the impact of the study by Kazaure et al. [21] on the final inference was explored with influence analysis, the summary estimate and heterogeneity decreased without affecting the final inference of the result (OR: 1.18; 95% CI: 0.62 to 1.75; *I*^2^: 0%; *P* < 0.0001). Egger's regression test (*P*=0.06127), Begg's rank test (*P*=1.000), inspection of the funnel plot, and fail-safe *N* test (*n* = 680) did not indicate any publication bias.

#### 3.2.4. Respiratory Disease

Six of the included studies reported on the incidence of preoperative respiratory disease (perioperative AF versus control: 2741 versus 107,821) [19, 21–24, 28]. The presence of respiratory disease was not a significant predictor of perioperative AF (perioperative AF versus control: 478 versus 9883; 17.4% versus 9.16%; OR: 1.20; 95% CI: −0.01 to 2.42; *I*^2^:77%; *P*=0.05). The preoperative respiratory disease included chronic obstructive pulmonary disease and chronic lung disease. Supplementary File S4 provides individual study data on the preoperative respiratory comorbidities in both the arrhythmia and control groups.

#### 3.2.5. Model for End-Stage Liver Disease (MELD) Score

Two studies involving 2446 patients reported the data on the MELD score [25, 26]. MELD score was not a significant predictor of perioperative AF in liver transplant patients. The average MELD score was 33.5 ± 2.8 (*n* = 115 patients) for the AF group versus 25.2 ± 9.1 (*n* = 2331 patients) for the control group (MD: 7.73; 95% CI: −0.93 to 16.4; *I*^2^: 87%; *P*=0.08). Influence analysis by excluding and including each study did not yield any significant difference in the pooled estimate.

#### 3.2.6. Type of Surgery

Supplementary Table S7 shows the surgery distribution for the perioperative AF and control groups. 93.20% of the patients with perioperative AF had abdominal/general surgery, followed by transplant (4.80%), vascular (1.2%), orthopaedic (0.51%), and head and neck surgery (0.24%). Similarly, in the control group, 96.69% of the patients had abdominal/general surgery, followed by transplant (2.35%), vascular (0.43%), orthopaedic (0.35%), and head & neck surgery (0.16%).

### 3.3. Postoperative Complications

**Figure 7:**
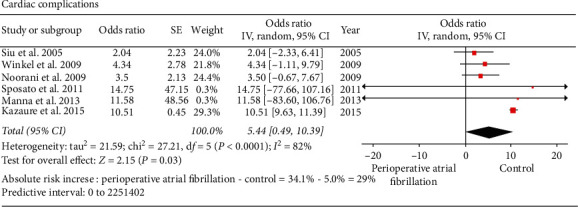
Forest plot comparing cardiac complications between atrial fibrillation group and control groups in patients undergoing noncardiothoracic surgery. The odds ratio of each included study is plotted. A pooled estimate of overall odds ratio (diamonds) and 95% confidence intervals (width of diamonds) summarizes the effect size using the random effects model. CI = confidence interval; M–H = Mantel–Haenszel; OR = odds ratio; *I*^2^: heterogeneity; *P* < 0.05 is significant.

Cardiac complications: six of the included studies reported on the incidence of postoperative cardiac complications (perioperative AF versus control: 2771 versus 107,885) [19, 21, 22, 24, 28, 29]. The occurrence of perioperative AF in patients undergoing noncardiothoracic surgery was significantly associated with postoperative cardiac complications (perioperative AF versus control: 973 versus 5432; 34.1% versus 5%; OR: 5.44; 95% CI: 0.49 to 10.39; *I*^2^: 82%; *P*=0.03) (Figure 7). Postoperative cardiac complications included myocardial infarction and congestive heart failure. Supplementary File S4 provides individual study data on the postoperative cardiac complications in both the AF and control groups. The study by Kazaure et al. [21] contributed maximum heterogeneity. When this study was excluded, the heterogeneity became 0%, and the final inference of the result did not change although the summary estimate decreased (OR: 3.18; 95% CI: 0.54 to 5.82; *I*^2^: 0%; *P*=0.02). Inspection of the funnel plot, Egger's test (*P*=0.11711), and Begg's test (*P*=1.0) did not indicate any publication bias.

Stroke: two studies involving 2686 patients in the AF group and 106586 patients in the control group reported the data on the stroke [21, 22]. Perioperative arrhythmia was significantly associated with postoperative stroke (perioperative arrhythmia vs Control: 15 versus 108; 0.5% versus 0.1%; OR: 3; 95% CI: 0.65 to 5.35; *I*^2^: 0%; *P*=0.01; not shown in the figure).

**Figure 8:**
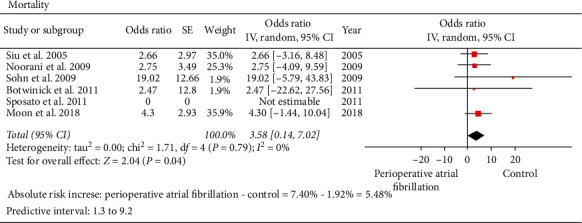
Forest plot comparing mortality between atrial fibrillation group and control groups in patients undergoing noncardiothoracic surgery. The odds ratio of each included study is plotted. A pooled estimate of overall odds ratio (diamonds) and 95% confidence intervals (width of diamonds) summarizes the effect size using the random effects model. CI = confidence interval; M–H = Mantel–Haenszel; OR = odds ratio; *I*^2^: heterogeneity; *P* < 0.05 is significant.

Mortality: six studies involving 108 patients in the AF group and 9905 patients in the control group reported the data on the mortality [19, 20, 22, 26–28]. Perioperative AF was a significant predictor of postoperative mortality. From the 6 studies reporting on mortality outcomes, 7.40% had perioperative mortality in arrhythmia group versus 1.92% in control group (perioperative AF versus control: 8 versus 191; OR: 3.58; 95% CI: 0.14 to 7.02; *I*^2^: 0%; *P*=0.04) (Figure 8). Inspection of the funnel plot, Egger's test (*P*=0.33536), Begg's test (*P*=0.80650), and fail-safe *N* test (*n* = 27) did not indicate any publication bias.

Study quality assessment: study quality assessment was conducted (as categorical variable) by meta-regression and sensitivity analysis of various subgroups based on the study type (retrospective versus prospective), quality of study (good versus poor-moderate), whether or not outcomes were clearly defined (yes versus no), sample size >1000 (yes versus no), and surgery type (transplant versus nontransplant procedures) did not show any significant differences in the results (Table 1).

## 4. Discussion

There is a change in the old perception that postoperative arrhythmias like AF were a benign condition [31, 32]. Newly diagnosed AF is identified as a risk factor for stroke, prolonged hospital stay, and hospitalization costs [21]. This SRMA of the risk factors and outcomes of perioperative AF associated with noncardiothoracic surgery identified advanced age, male gender, preexisting cardiac comorbidities, hypertension, and diabetes mellitus as the risk factors associated with perioperative AF. The AF group had increased odds for perioperative cardiac complications, stroke, and mortality compared to the non-AF group.

Evidence on the incidence of postoperative arrhythmias shows that 16% to 46% of patients after cardiac surgery, 3% to 30% of patients after thoracic surgery, and up to 8% of noncardiothoracic surgical patients developed new-onset atrial arrhythmias [33]. Evidence on the incidence of AF after noncardiothoracic surgery is largely variable. From the studies included in our analysis, we calculated an incidence of 2.49%. Sohn et al. [27] reported that 0.39% of patients experienced postoperative atrial fibrillation (POAF) after noncardiothoracic surgery, similar to the 0.37% found by Christians et al. [34], abdominal surgery is associated with a higher incidence of perioperative AF versus other noncardiothoracic surgical procedures [35]. Kazaure et al. [21] found that 1 in 8 patients over 65 years, and 1 in 4 patients over 85 years, had AF after abdominal surgery. This is also confirmed by our analysis which showed that 90.87% of the patients in the arrhythmia group had abdominal or general surgery. A literature review and analysis [36] showed that the majority of perioperative arrhythmias are supraventricular in origin and AF was the single most common arrhythmia.

Recognizing the risk factors of perioperative AF helps to individualize patient care, as well as to guide future studies of interventions to lower the incidence of perioperative AF and the associated complications. Age is an important predictor of perioperative AF. Our study found that the perioperative AF group patients were older compared to the control group. This is consistent with the findings from other studies [20, 24, 27]. Three studies have identified age as an independent predictor for perioperative AF in a multivariate analysis [25, 27, 29]. A prospective observational study of noncardiothoracic surgical patients [37] found that the average age of patients with new onset of atrial arrhythmias was around 67 years. But this study reported on atrial flutter, paroxysmal supraventricular tachycardia, and multifocal atrial tachycardia, in addition to AF. Likewise, Manna et al. [29] identified an age cut-off value of 53 years for the incidence of POAF after renal transplant surgery, but the authors agree that their study was inadequately powered to identify a real cut-off threshold of AF in this patient population. A study found that female sex was associated with a lower-risk of postoperative AF [21], which is in agreement with the findings of this meta-analysis.

We identified hypertension and diabetes as predictors of perioperative arrhythmias. Hypertension is well established to be a risk factor for perioperative AF in both experimental animal and human studies [38, 39]. The probable hemodynamic mechanisms include the increase in left ventricular wall thickness, stiffness, that may lead to a rise in left atrial stretch and pressure, and subsequent remodeling, ultimately predisposing to AF [40]. Studies have indicated that inflammation associated with diabetes might play a role in the pathophysiology of AF and a multivariate analysis from a community study showed that DM is independently associated with AF (OR: 1.46) [41].

In our review, increased BMI was not identified as a risk factor for perioperative AF. However, BMI was identified as an important risk factor of new-onset atrial fibrillation (NOAF) after cardiac surgery [42]. Interestingly, Sohn et al.'s study [27] of noncardiothoracic surgery patients found the opposite result, as the patients with AF had a significantly lower BMI versus patients without AF. Although additional studies are required, the authors have attributed this to the obesity paradox, which holds that obesity may in fact be protective and associated with greater survival in certain groups, like the elderly [43].

In our analysis, preexisting cardiac disease increases the odds of perioperative AF. In a study by Christians et al. [34], of POAF in noncardiothoracic surgical patients, at least one cardiac risk factor was found in 67% percent of the patients. This study did not include a control group and hence was not included in our analysis.

Fulminant hepatic failure and a higher MELD score were identified as independent predictors of intraoperative AF [25, 26]. In our study, the MELD score was not a significant predictor of perioperative AF in liver transplant patients. The sympathetic hyperfunction and autonomic imbalance is attributable to the heart rate variability changes in patients with liver failure [44].

Perioperative AF after noncardiothoracic surgery has been associated with poor postoperative cardiovascular outcomes. Our meta-analysis found that perioperative AF was associated with increased odds of postoperative cardiac complications, like myocardial infarction (MI) and congestive cardiac failure. Four studies have identified perioperative AF as an independent predictor for postoperative cardiac complications in a multivariate analysis [19, 21, 24, 29]. NOAF was found to be associated with MI and stroke after carotid endarterectomy [22]. A retrospective study on patients undergoing aortic repair by Noorani et al. [19] found that NOAF was associated with a greater risk of MI, which should be seriously considered in case of a NOAF after surgery involving the aorta. Our study also found that cardiac failure was independently associated with AF. In another study by Winkel et al. [24], a strong association between AF and myocardial ischemia was seen, as evidenced by an increased release of troponin T. However, the temporal relationship between myocardial ischemia and AF could not be proved as troponin T was only measured at intervals. Furthermore, myocardial ischemia preceded the onset of NOAF in about half of the cases.

Our study found increased odds of mortality in the AF group versus the control group. Several other studies [33, 45], examining AF following noncardiothoracic surgery reported a mortality increase. Two studies have identified perioperative AF as an independent predictor for postoperative mortality in a multivariate analysis [21, 26]. Our finding is also consistent with the findings of Leibowitz et al. [23] who reported a significant increase in one-year mortality in patients with AF versus those without AF after hip fracture surgery (60% versus 19.5%; *P*=0.001) that is not attenuated by antiarrhythmic therapy to treat AF. There are several reasons attributed to this increased mortality in AF patients after hip fracture surgery. The loss of effective atrial contraction secondary to AF is associated with hemodynamic decompensation and thromboembolic events [46, 47]. Elderly patients are particularly dependent on atrial contraction for ventricular filling as they commonly suffer from noncompliant left ventricles and are more vulnerable to this complication of AF [48, 49]. Similarly, another study reported a significantly higher one-year mortality in elderly adults with atrial arrhythmias after surgery for hip fracture, but this study included patients with preoperative AF [50]. Christians et al. [34] reported a one-month mortality of 12% in AF patients after noncardiothoracic surgery. In another study of oncological surgical procedures, the in-hospital mortality and long-term mortality were 14% and 41%, respectively [51]. But it is noteworthy that no control population was included in both the above studies. Intraoperative AF was found to be an independent predictor of postoperative mortality with a 4.5 times higher risk of mortality after liver transplantation, despite its low incidence (1.2%) and short duration (1 hour) [26]. This is attributed to the increased stress on the cardiovascular system, with large perioperative fluid shifts, hemodynamic changes, and also because of the aggressive treatment of intraoperative hemorrhage with aggressive vasopressor and fluid therapy [52]. A subgroup analysis also allowed us to shed light on the group of transplant surgical patients: it showed an increased mortality rate and postoperative cardiac complications in the arrhythmia group, regardless of the presence of other risk factors such as advanced age, history of cardiac disease, and hypertension which were not significant in this population.

### 4.1. Limitations

The lack of consistent definition, precision in identifying AF, and consistent monitoring protocols are major risks for bias in many of the included studies. The application of cardiac monitoring intraoperatively was reported in only 2 studies [22, 26], although it may be assumed that all patients are likely to have continuous monitoring in the intraoperative period. Three studies [19, 22, 24] applied continuous cardiac monitoring for at least 72 hours, and four studies [23, 25, 27, 28] reported conducting “regular” ECGs in the postoperative setting. The second limitation is that although studies included in our SRMA did not include patients with preoperative AF, not every study would have screened the patients for preoperative AF. Third, studies included in this SRMA were observational studies with a higher likelihood of intrinsic bias and may have contributed to a high degree of heterogeneity in our results. Fourth, the confounding effect on the risk factors is another limitation. Although the majority of the included studies performed multivariate regression analysis to correct for confounding variables, we were unable to pool the adjusted risk estimates and outcomes as they were sparsely reported for all the prognostic factors and the studies greatly differed in the number and the type of prognostic factors that were adjusted in the regression analyses. Furthermore, the causality relationship between perioperative AF and postoperative complications may not be conclusive owing to the retrospective nature of majority of the included studies. However, our meta-regression analysis would help in overcoming these biases to some extent. Lastly, even though publication bias was ruled out by multiple tests (Egger's regression test, Begg's rank test, and fail-safe *N* test), complete absence of publication bias cannot be ruled out. Hence, a well-designed prospective study with uniform definition, monitoring and follow-up for AF would help to perform risk stratification and to identify the outcomes and prognosis of the patients with perioperative AF.

## 5. Conclusion

This SRMA identified advanced age, male gender, preexisting hypertension, diabetes mellitus, and cardiac disease as important risk factors associated with perioperative AF. We also found that the AF group had increased odds for postoperative cardiac complications, stroke, and higher mortality compared to the control group, emphasizing the need for risk stratification and close monitoring of this surgical population.

## Figures and Tables

**Table 1:** Study quality assessment: meta-regression and sensitivity analysis of various subgroups (as categorical variable).

Risk factor or outcome	Study characteristics (number of studies)	Summary estimate	95% CI	*I* ^2^	Meta-regression	
Age	Coefficient (SE)	*P* value
Study type	Retrospective (9) [19–23, 26–29]	3.75	0.75–6.76	90	0.6155	0.8651
	Prospective (2) [24, 25]	5.03	2.97–7.09	0	(3.6220)	
Quality of study	Poor-moderate (1) [20]	14.34	−11.37–40.05	—	−0.3148	0.9486
	Good (10) [19, 20–29]	3.97	1.57–6.38	89	(4.8794)	
Outcome defined	Yes (7) [19, 21, 22, 24, 25, 27, 28]	4.58	2.08–7.08	88	0.3634	0.9250
	No (4) [20, 23, 26, 29]	3.12	−4.31–10.55	83	(3.8607)	
Sample size >1000	Yes (4) [21, 25–27]	3.11	−0.41–6.62	94	1.9924	0.4748
	No (7) [19, 20, 22–24, 28, 29]	4.93	1.51–8.34	67	(2.7880)	
Surgery type	Transplant (3) [25, 26, 29]	3.04	−4.03–10.11	90	−1.6709	0.6740
	Nontransplant (8) [19–24, 27, 28]	4.42	1.68–7.17	86	(3.9716)	

Gender						
Study type	Retrospective (9) [19–23, 26–29]	1.07	0.42–1.72	87	−0.1621	0.8230
	Prospective (2) [24, 25]	1.15	0.66–1.64	0	(0.7248)	
Quality of study	Poor-moderate (1) [20]	0.68	−0.26–1.62	—	−0.5386	0.5592
	Good (10) [19, 21–29]	1.14	0.55–1.72	86	(0.9223)	
Outcome defined	Yes (7) [19, 21, 22, 24, 25, 27, 28]	1.43	1.33–1.52	0	0.4614	0.5366
	No (4) [20, 23, 26, 29]	0.56	−0.01–1.12	34	(0.7465)	
Sample size >1000	Yes (4) [21, 25–27]	1.07	0.29–1.86	95	−0.3247	0.4981
	No (7) [19, 20, 22–24, 28, 29]	1.02	0.46–1.57	0	(0.4793)	
Surgery type	Transplant (3) [25, 26, 29]	0.80	−0.04–1.63	83	−0.3795	0.5944
	Nontransplant (8) [19–24, 27, 28]	1.43	1.33–1.53	0	(0.7126)	

Hypertension						
Study type	Retrospective (8) [19, 21–23, 26–29]	1.15	1.07–1.22	0	−0.1036	0.8490
	Prospective (2) [24, 25]	1.24	0.75–1.74	0	(0.5441)	
Quality of study	Poor-moderate (0)	—	—	—	—	—
	Good (10) [19, 21–29]	1.15	1.08–1.23	0		
Outcome defined	Yes (7) [19, 21, 22, 24, 25, 27, 28]	1.15	1.08–1.23	0	−0.8519	0.1879
	No (3) [23, 26, 29]	2.81	−0.77–6.39	0	(0.6469)	
Sample size >1000	Yes (4) [21, 25–27]	1.15	1.08–1.23	0	−0.2608	0.3738
	No (6) [19, 22–24, 28, 29]	1.32	0.50–2.13	0	(0.2933)	
Surgery type	Transplant (3) [25, 26, 29]	1.24	0.74–1.75	0	−0.0418	0.9409
	Nontransplant (7) [19, 21–24, 27, 28]	1.15	1.07–1.23	0	(0.5639)	

Cardiac disease						
Study type	Retrospective (7) [19, 21–23, 26, 28, 29]	2.79	0.17–5.41	92	1.3910	0.0545
	Prospective (2) [24, 25]	1.32	0.16–2.49	55	(0.7236)	
Quality of study	Poor-moderate (0)	—	—	—	—	—
	Good (9) [19, 21–26, 28, 29]	2.30	0.28–4.31	93		
Outcome defined	Yes (6) [19, 21, 22, 24, 25, 28]	2.15	−0.16–4.46	95	0.0043	0.9956
	No (3) [23, 26, 29]	3.28	−1.90–8.46	27	(0.7790)	
Sample size >1000	Yes (3) [21, 25, 26]	3.86	0.90–6.82	92	0.7579	0.1808
	No (6) [19, 22–24, 28, 29]	0.94	0.29–1.58	0	(0.5663)	
Surgery type	Transplant (3) [25, 26, 29]	2.94	−0.63–6.52	13	0.9274	0.2304
	Nontransplant (6) [19, 21–24, 28]	2.01	−0.42–4.44	96	(0.7732)	

Diabetes mellitus						
Study type	Retrospective (8) [19, 21–23, 26–29]	0.97	0.89–1.05	0	0.7277	0.4019
	Prospective (1) [24]	0.61	−0.70–1.92	—	(0.8681)	
Quality of study	Poor-moderate (0)	—	—	—	—	—
	Good (9) [19, 21–24, 26–29]	0.97	0.89–1.05	0		
Outcome defined	Yes (6)[19, 21, 22, 24, 27, 28]	0.97	0.89–1.05	0	−0.2032	0.7579
	No (3)[23, 26, 29]	1.51	0.14–2.88	0	(0.6595)	
Sample size >1000	Yes (3)[21, 26, 27]	0.97	0.89–1.05	0	−0.2644	0.5052
	No (6) [19, 22–24, 28, 29]	0.94	0.17–1.71	0	(0.3968)	
Surgery type	Transplant (2) [26, 29]	1.48	−0.51–3.48	0	0.3992	0.5901
	Nontransplant (7) [19, 21–24, 27, 28]	0.97	0.89–1.05	0	(0.7410)	

Cardiac complications						
Study type	Retrospective (5) [19, 21, 22, 28, 29]	5.71	−0.18–11.61	83	0.3609	0.6949
	Prospective (1) [24]	4.34	−1.11–9.79	—	(0.9292)	
Quality of study	Poor-moderate (0)	—	—	—	—	—
	Good (6) [19, 21, 22, 24, 28, 29]	5.44	0.49–10.39	82		
Outcome defined	Yes (5) [19, 21, 22, 24, 28]	5.41	0.36–10.46	85	−0.7377	0.6474
	No (1) [29]	11.58	−83.6–106.76	0	(1.6127)	
Sample size >1000	Yes (1) [21]	10.51	9.63–11.39	—	0.9718	0.0023
	No (5) [19, 22, 24, 28, 29]	3.18	0.54–5.82	0	(0.3193)	
Surgery type	Transplant (1) [29]	11.58	−83.60–106.76	—	0.7377	0.6474
	Nontransplant (5) [19, 21, 22, 24, 28]	5.41	0.36–10.46	85	(1.6127)	

Mortality						
Study type	Retrospective (6) [19, 20, 22, 26–28]	3.58	0.14–7.02	0	—	—
	Prospective (0)	—	—	—		
Quality of study	Poor-moderate (1) [20]	2.47	−22.62–27.56	—	−0.7977	0.6530
	Good (5) [19, 22, 26–28]	3.60	0.13–7.08	0	(1.7743)	
Outcome defined	Yes (4) [19, 22, 27, 28]	3.20	−1.16–7.57	0	0.4607	0.6670
	No (2) [20, 26]	4.21	−1.39–9.81	0	(1.0705)	
Sample size >1000	Yes (2) [26, 27]	6.51	−3.79–16.81	22	1.2296	0.0934
	No (4) [19, 20, 22, 28]	2.69	−1.67–7.06	0	(0.7329)	
Surgery type	Transplant (1) [26]	4.30	−1.44–10.04	—	−0.2072	0.8651
	Nontransplant (5) [19, 20, 22, 27, 28]	3.18	−1.12–7.48	0	(1.2196)	

Study quality scores were obtained from the Ottawa–Newcastle quality assessment [16]. Study was considered good when assigned score was equal 9. For respiratory complications and Stroke, meta-regression analysis not conducted due to inadequate number of studies.

## Data Availability

The data are available upon request to the corresponding author.

## Abbreviations

AF: Atrial Fibrillation
BMI: Body mass index
CI: Confidence Interval
CMA: Comprehensive Meta-Analysis
ECG: Electrocardiogram
MD: Mean Difference
MELD: Model for End-stage Liver Disease
NOAF: New-Onset Atrial Fibrillation
OR: Odds Ratios
PRISMA: Preferred Items for Systematic Reviews and Meta-Analyses
QUIPS: Quality in Prognostic Studies
SRMA: Systematic Review and Meta-analysis

## Supplementary Materials

Supplementary Materials S1: predesigned study protocol. S2: study search strategy. S3: definitions of outcome atrial fibrillation. S4: table on systematic review. S5: Newcastle–Ottawa scale scoring system (quantitative study assessment). S6: bias assessment results of each study with the quality in prognostic studies (QUIPS) tool. S7: distribution of the type of surgical procedures for the atrial fibrillation group and control groups.
