# Supplementary material for: Incidence, Risk Factors, and Outcomes of Perioperative Atrial Fibrillation following Noncardiothoracic Surgery: A Systematic Review and Meta-Regression Analysis of Observational Studies
Source: Anesthesiol Res Pract. 2021 Apr 28;2021:5527199. doi: 10.1155/2021/5527199 (PMC8099514; doi:10.1155/2021/5527199)
Supplement: Supplementary Materials — S1: predesigned study protocol. S2: study search strategy. S3: definitions of outcome atrial fibrillation. S4: table on systematic review. S5: Newcastle–Ottawa scale scoring system (quantitative study assessment). S6: bias assessment results of each study with the quality in prognostic studies (QUIPS) tool. S7: distribution of the type of surgical procedures for the atrial fibrillation group and control groups. [file 5527199.f1.docx]

**Supplementary file:**

Table of Contents

[S1 – Predesigned Study protocol 2](#_Toc68727108)

[S2 – Study search strategy 7](#_Toc68727109)

[S3 – Definitions of outcome Atrial Fibrillation 12](#_Toc68727110)

[S4 – Table on Systematic Review 14](#_Toc68727111)

[S5 – Newcastle-Ottawa scale scoring system (Quantitative study assessment) 21](#_Toc68727112)

[S6 – Bias assessment results of each study with the Quality in Prognostic Studies (QUIPS) tool 23](#_Toc68727113)

[S7 – Distribution of the type of surgical procedures for the arrhythmia and control groups 26](#_Toc68727114)

# **S1 –** Predesigned Study protocol

**Title:**

Incidence, risk factors and management of perioperative atrial fibrillation following non-cardiothoracic surgery. A Systematic Review and Meta-analysis

**Objective:**

In this systematic review and meta-analysis, we aim to investigate the incidence, risk factors and outcomes associated with the occurrence of perioperative arrhythmias following non-cardiothoracic surgery.

**Inclusion criteria:**

Study type

- All observational cohort studies will be eligible to enter meta-analysis.
- We will include all studies which were published as original reports and present information on on cases of new-onset postoperative cardiac arrhythmias after non-cardiothoracic surgery along with a non-arrhythmia (control) group.

Participants

- Adult patients (>18 years) of all age groups undergoing surgery will be included.

Outcome measures

- The OR will either be extracted from the published article or calculated by the authors.
- If the OR is not directly reported or cannot be readily extracted from the published data, the reviewers will contact the corresponding authors for additional information (e.g., data provided in 2x2 contingency tables).

Publication type

- Full published papers will be eligible.

**Exclusion criteria:**

- Studies without explicit reporting of postoperative cardiac arrhythmias will be excluded.
- Studies on cardio-thoracic surgery will be excluded.
- Case reports, case series, abstracts, conference proceedings and non-english language studies will be excluded.

**Search Methods:**

We will search the following electronic databases:

- MedLine (via PubMed)
- EMBASE
- Web of Science
- Cochrane CENTRAL Register of Controlled Trials and Cochrane Database of Systematic Reviews
- The search will be restricted to English language. In these databases, we will search according to the thesaurus of the NCBI MESH browser the following terms and combinations of keywords in full text:

The following keywords will be employed:

The search included the combination of the following MESH key words: ("after surgery" or "following surgery or post-surgery or post-transplant or perioperative or periprocedural or intraoperative or intraprocedural or postoperative, postprocedural, perioperative period, arrhythmia or bradycardia or tachycardia or Auricular fibrillation or afib or dysrhythmia or tachyarrhythmia or bradyarrhythmia.

Additionally, bibliographies of identified publications and published reviews will be hand searched for potentially relevant articles. Authors will be contacted if data, methods and/or parameter definitions provided from the respective studies are unclear. All references cited in the identified reviews will be manually searched for potentially relevant studies.

**Data collection:**

Three reviewers (OE, DJ, YS) will independently scrutinize the list of titles, and if available the abstracts, to determine potential usefulness of the article. Final selection will be based on the full text of potentially relevant articles by the two reviewers independently. In cases of disagreement senior author (FA) would help in achieving a consensus. Study quality will be measured using the modified version of Newcastle–Ottawa scale^1^

The following study characteristics will be extracted: study ID, publication year, country, study design, cohort size, demographic data (age, gender, BMI), comorbidities, surgical procedures and perioperative complications. From all eligible studies, relevant data will be abstracted in duplicate, using a standardized data extraction sheet. A senior author (YS) will confirm all data entries and will check at least twice for completeness and accuracy.

**Meta-analysis & Meta-regression:**

Dichotomous comparisons

- Data on numbers of comorbidities and perioperative complications and corresponding crude odds ratios and 95% confidence intervals in the 2 groups will be calculated.
- Random-effects models to estimate the pooled odds ratios for risk of perioperative complications in both the groups will be constructed across all studies. The value of the I^2^ statistic will be used to select the appropriate pooling method: fixed-effects models were used for I^2^<50% and random-effects models for I^2^>=50%.

Assessment of heterogeneity:

- Impact of heterogeneity will be assessed by calculating the I^2^ according to Higgins et al^2^. Confidence intervals around the I^2^ were also provided.

Influence analysis:

- Robustness of the pooled estimates will be checked by influence analyses. Each of the studies will be individually omitted from the data set, followed in each case by recalculation of the pooled estimate of the remaining studies.

Subgroup/Sensitivity analyses:

- To identify potential sources of heterogeneity and sources of bias, studies will be stratified by study design, quality of the study, study quality scores, measured outcome definitions and loss of patients to follow-up
- Forest plots: Forest plots will be re-plotted with summary points.

**Evaluation of bias and confounding**

Publication bias

- Publication bias will be assessed by inspection of the funnel plot and formal testing for

funnel plot asymmetry.

Funnel plot in inverse-V shape will be presented.

**Discussion and Evaluating:**

- The results will be critically and integratively discussed.

**References:**

1. Wells G, Shea B, O’connell D, Peterson J, … VW-EA from: U, 2016 undefined. The Newcastle-Ottawa Scale (NOS) for assessing the quality if nonrandomized studies in meta-analyses. 2009.

2. Higgins JPT, Thompson SG, Deeks JJ, Altman DG. Measuring inconsistency in meta-analyses. BMJ 2003;327:557–60.

# **S2 –** Study search strategy

Rachel Sandieson

Rachel.Sandieson@lhsc.on.ca

Ext. 35134

UH B3-406

**Systematic Review – Search Notes**

Date: February 4, 2019

For: Ashraf Fayad

Subject: Postoperative arrhythmias; treatment and outcomes

Databases Used: Medline, Embase, Cochrane CENTRAL Register of Controlled Trials, Cochrane Database of Systematic Reviews, Web of Science

Notes:

**Notes:**

- Final searches were run on February 1, 2019
- Please see accompanying RIS file
- Note that duplicates have been removed.

**Results by Database:**

| **Database** | **Results** |
| --- | --- |
| Medline & Medline in-process | 1346 |
| Embase | 558 |
| Cochrane CENTRAL Register of Controlled Trials and Cochrane Database of Systematic Reviews | 694 |
| Web of Science | 5 |
|  |  |
| *Duplicates Removed* | *370* |
|  |  |
| **Total** | **2233** |

**Search Strategies:**

Medline

Database(s): **Ovid MEDLINE(R) ALL**1946 to January 31, 2019 
Search Strategy:

| **#** | **Searches** | **Results** |
| --- | --- | --- |
| 1 | ("after surg*" or "following surg*" or post?surg* or post?transplant* or peri?operat* or peri?proced* or intra?operat* or intra?proced* or post?opt*).ti,ab. | 396457 |
| 2 | exp Perioperative Period/ | 79855 |
| 3 | or/1-2 | 448690 |
| 4 | (arrhythmia* or bradycard* or tachycard* or Auricular fibrillation or afib or dysrhythmia* or tachyarrhythmia* or bradyarrhythmia*).ti,ab. | 145260 |
| 5 | ((atrial or atrium or auricular or ventric*) and (flutter* or fibrillat*)).ti,ab. | 83349 |
| 6 | ((abnormal or disorder) and rhythm).ti,ab. | 4603 |
| 7 | exp Arrhythmias, Cardiac/ | 196758 |
| 8 | or/4-7 | 285010 |
| 9 | (treatment* or Anti-Arrhythmia Agent* or drug* or agent* or therap* or intervention* or medication*).ti,ab. | 7186704 |
| 10 | exp therapeutics/ | 4295104 |
| 11 | or/9-10 | 9486387 |
| 12 | (outcome* or effect* or risk* or mortality or death or hospitaliz* or "follow up*" or survival or morbidity or complication* or failure or success or recur* or progress* or score).ti,ab. | 10881367 |
| 13 | exp Treatment Outcome/ | 953770 |
| 14 | (postoperative complications or periperative complications).sh. | 342362 |
| 15 | or/12-14 | 11193910 |
| 16 | 3 and 8 and 11 and 15 | 7113 |
| 17 | limit 16 to english language | 6121 |
| 18 | limit 17 to "all adult (19 plus years)" | 4008 |
| 19 | (case reports or editorial or abstracts).pt. | 2399739 |
| 20 | 18 not 19 | 3585 |
| 21 | exp cardiac surgical procedures/ | 203841 |
| 22 | ((cardiac or heart) and (surg* or procedure*)).ti,ab. | 181366 |
| 23 | 21 or 22 | 326618 |
| 24 | 20 not 23 | 1346 |

Embase

Database(s): **Embase Classic+Embase**1947 to 2019 January 31 
Search Strategy:

| **#** | **Searches** | **Results** |
| --- | --- | --- |
| 1 | ("after surg*" or "following surg*" or post?surg* or post?transplant* or peri?operat* or peri?proced* or intra?operat* or intra?proced* or post?opt*).ti,ab. | 557553 |
| 2 | exp Perioperative Period/ | 43882 |
| 3 | or/1-2 | 568694 |
| 4 | (arrhythmia* or bradycard* or tachycard* or Auricular fibrillation or afib or dysrhythmia* or tachyarrhythmia* or bradyarrhythmia*).ti,ab. | 225488 |
| 5 | ((atrial or atrium or auricular or ventric*) and (flutter* or fibrillat*)).ti,ab. | 143406 |
| 6 | ((abnormal or disorder) and rhythm).ti,ab. | 8506 |
| 7 | exp heart arrhythmia/ | 456730 |
| 8 | or/4-7 | 555215 |
| 9 | (treatment* or Anti-Arrhythmia Agent* or drug* or agent* or therap* or intervention* or medication*).ti,ab. | 10134087 |
| 10 | exp therapy/ | 8224590 |
| 11 | or/9-10 | 13906161 |
| 12 | (outcome* or effect* or risk* or mortality or death or hospitaliz* or "follow up*" or survival or morbidity or complication* or failure or success or recur* or progress* or score).ti,ab. | 14649527 |
| 13 | exp Treatment Outcome/ | 1487376 |
| 14 | exp postoperative complication/ | 646563 |
| 15 | perioperative complication/ | 21477 |
| 16 | or/12-15 | 15149839 |
| 17 | 3 and 8 and 11 and 16 | 15343 |
| 18 | limit 17 to english language | 14088 |
| 19 | limit 18 to (adult <18 to 64 years> or aged <65+ years>) | 7670 |
| 20 | (case reports or editorial or abstracts).pt. | 596105 |
| 21 | 19 not 20 | 7667 |
| 22 | exp heart surgery/ | 352344 |
| 23 | ((cardiac or heart) and (surg* or procedure*)).ti,ab. | 290891 |
| 24 | or/22-23 | 526715 |
| 25 | 21 not 24 | 3572 |

Cochrane CENTRAL Register of Controlled Trials and Cochrane Database of Systematic Reviews

Database(s): **EBM Reviews - Cochrane Central Register of Controlled Trials**December 2018**, EBM Reviews - Cochrane Database of Systematic Reviews**2005 to January 30, 2019 
Search Strategy:

| **#** | **Searches** | **Results** |
| --- | --- | --- |
| 1 | ("after surg*" or "following surg*" or post?surg* or post?transplant* or peri?operat* or peri?proced* or intra?operat* or intra?proced* or post?opt*).ti,ab. | 150723 |
| 2 | exp Perioperative Period/ | 7698 |
| 3 | or/1-2 | 152838 |
| 4 | (arrhythmia* or bradycard* or tachycard* or Auricular fibrillation or afib or dysrhythmia* or tachyarrhythmia* or bradyarrhythmia*).ti,ab. | 12632 |
| 5 | ((atrial or atrium or auricular or ventric*) and (flutter* or fibrillat*)).ti,ab. | 9837 |
| 6 | ((abnormal or disorder) and rhythm).ti,ab. | 405 |
| 7 | exp Arrhythmias, Cardiac/ | 8434 |
| 8 | or/4-7 | 22108 |
| 9 | (treatment* or Anti-Arrhythmia Agent* or drug* or agent* or therap* or intervention* or medication*).ti,ab. | 783730 |
| 10 | exp therapeutics/ | 274216 |
| 11 | or/9-10 | 861826 |
| 12 | (outcome* or effect* or risk* or mortality or death or hospitaliz* or "follow up*" or survival or morbidity or complication* or failure or success or recur* or progress* or score).ti,ab. | 904146 |
| 13 | exp Treatment Outcome/ | 122555 |
| 14 | (postoperative complications or perioperative complications).sh. | 15792 |
| 15 | or/12-14 | 920085 |
| 16 | 3 and 8 and 11 and 15 | 2666 |
| 17 | limit 16 to english language [Limit not valid in CDSR; records were retained] | 2067 |
| 18 | (case reports or editorial or abstracts).pt. | 2021 |
| 19 | 17 not 18 | 2064 |
| 20 | exp cardiac surgical procedures/ | 12157 |
| 21 | ((cardiac or heart) and (surg* or procedure*)).ti,ab. | 23384 |
| 22 | 20 or 21 | 30754 |
| 23 | 19 not 22 | 694 |

Web of Science

(TI=("after surg*") OR TI=("following surg*") OR TI=(post?surg*) OR TI=(post?transplant*) OR TI=(peri?operat*) OR TI=(peri?proced*) OR TI=(intra?operat*) OR TI=(intra?proced*) OR TI=(post?opt*))

9,787

(((TI=arrhythmia*) OR TI=(bradycard*) OR TI=(tachycard*) OR TI=(Auricular fibrillation) OR TI=(afib) OR TI=(dysrhythmia*) OR TI=(tachyarrhythmia*) OR TI=(bradyarrhythmia*)) OR (((TI=atrial) OR TI=(atrium) OR TI=(auricular) OR TI=(ventric*)) AND (TI=flutter*) OR TI=(fibrillat*)) OR ((TI=abnormal) OR TI=(disorder)) AND (TI=(rhythm)))

51,902

(TI=(treatment*) OR TI=(Anti-Arrhythmia Agent*) OR TI=(drug*) OR TI=(agent*) OR TI=(therap*) OR TI=(intervention*) OR TI=(medication*))

1,422,179

(TI=(outcome*) OR TI=(effect*) OR TI=(risk*) OR TI=(mortality) OR TI=(death) OR TI=(hospitaliz*) OR TI=("follow up*") OR TI=(survival) OR TI=(morbidity) OR TI=(complication*) OR TI=(failure) OR TI=(success) OR TI=(recur*) OR TI=(progress*) OR TI=(score))

3,874,945

# S3 – Definitions of outcome Atrial Fibrillation

| **Article** | **Type of arrhythmia** | **Definition** | **Method of Detection** | **Onset** |
| --- | --- | --- | --- | --- |
| ^26^Moon et al. ^2018^ | IOAF | No definition | Intraoperative full-scale hemodynamic data were recorded by using a computerized data acquisition system (DI-720U, DATAQ Instruments, Inc., Akron, OH, United States) [10]. Development of IOAF was detected by retrospective review of this data acquisition system data. Onset time, duration, and prodromal arrhythmia were also noted if IOAF developed | Intraoperative |
| ^23^Leibowitz et al. ^2017^ | NOAF | No definition | Diagnosis was made using standard electrocardiographic criteria by an experienced cardiologist | Postoperative |
| ^21^Kazaure et al. ^2015^ | NOAF | AF was defined by the presence of ICD-9-CM diagnosis code 427.3x | Presence of ICD-9-CM diagnosis code 427.3x | Timing not reported |
| ^25^Xia et al. ^2015^ | POAF | POAF was defined as new onset AF during LT surgery or within 30 days after LT in a patient who present no AF at the time of LT surgery | Diagnosis of AF was confirmed by the electrocardiographic tracings, reports, and notes of anesthesiologist, cardiologist and the ICU team | 80% within first Postoperative week |
| ^29^Manna et al. ^2013^ | POAF | No definition | Review of clinical charts. | 4.72 +/-5.75 Postoperative Median – 3 days |
| ^20^Botwinick et al. ^2011^ | AF | No definition | Retrospective chart review | Postoperative |
| ^22^Sposato et al. ^2011^ | NOAF | AF was defined by the absence of p waves or evidence of atrial flutter with irregular ventricular response | Patients underwent continuous electrocardiograph monitoring during surgery and throughout hospital stay | 4.0 +/-2.1 Postoperative days |
| ^27^Sohn et al. ^2009^ | POAF | Postoperative atrial fibrillation was defined as a new onset of atrial fibrillation after surgery and during hospitalization | The medical records, including the pre- and post-operative electrocardiograms, vital signs, consult sheets, discharge summaries, nursing information, and intensive care unit records of the patients were reviewed and determined whether the atrial fibrillation was pre-existing or new onset | Median – 2 days Postoperative. Lowest – 1 day Highest – 12 days |
| ^24^Winkel et al. ^2009^ | NOAF | On the ECG, AF is characterised by the replacement of consistent P waves with rapid oscillations or fibrillatory waves that vary in amplitude, shape and timing, associated with an irregular, frequently rapid, ventricular response when atrioventricular conduction is intact | Continuous-ECG recordings for 72 h and standard ECG on days 3, 7 and 30 were used to identify new-onset AF | All – POAF (Day 3-7) One- IOAF |
| ^19^Noorani et al. ^2009^ | NOAF | New-onset AF was detected on cardiac monitors was confirmed by 12-lead ECG | All patients underwent continuous electrocardiogram monitoring for the first 24 h postoperatively, in either the intensive care unit or a dedicated ‘FastTrack’ recovery bed. Cardiac monitoring was continued until the patient was discharged to the general ward | 100% within Postoperative Week Median: 4 Post-Op days |
| ^28^Siu et al. ^2005^ | POAF | Postoperative AF was defined as sustained AF episodes documented by electrocardiogram, which lasted >10 minutes during the in-hospital stay | Postoperative ECG | 76% of POAF occurred >2 Post-Op days |

NOAF – New Onset Atrial Fibrillation; POAF- Postoperative Atrial Fibrillation; MAT – Multifocal Atrial Tachycardia; AF – Atrial Fibrillation

# S4 – Table on Systematic Review

| **Study ID**  **(First Author, Year, Reference)** | **Demographics &**  **Comorbidities** | **Surgical duration &**  **Post-operative outcomes** |
| --- | --- | --- |
| ^26^Moon et al. ^2018^ | • Arrhythmias vs. Control  13 vs.1046  • Age:  45.4 (8.7) vs. 50.6 (8.7)  • Sex (male):  38.4% vs. 74.5%  • Hypertension:  23% vs. 10.7%  • Diabetes Mellitus:  23% vs. 19.3%  • Cardiac Disease:  46.1% vs. 7.8%  • MELD Score:  31.5 (11.2) vs. 18.8 (9.8) | • Surgery Duration:  749.6 (124.7) vs. 862.3 (163.3)  • Mortality:  3.4% vs. 16.6% |
| ^23^Leibowitz et al. ^2017^ | • Arrhythmias vs. Control  15 vs. 395  • Age:  82.8 (6.6) vs. 80 (7.9)  • Sex (male):  40% vs. 33.6%  • Hypertension:  86.6% vs. 64.8%  • Diabetes Mellitus:  46.6% vs. 36.2%  • Cardiac Disease:  IHD: 40% vs. 25%  Heart Failure: 6.6% vs. 8.8%  Valvular: 0 vs. 7.5%  • Respiratory Disease:  COPD 6.6% vs. 5%  • History of AF:  33.3% vs. 8.6% |  |
| ^21^Kazaure et al. ^2015^ | • Arrhythmias vs. Control  2679 vs. 106407  • Age:  75.0 (0.1) vs. 67.9 (0.0)  • Sex (male):  51.7% vs. 42.6%  • Hypertension:  61.1% vs. 57.9%  • Diabetes Mellitus:  23.0% vs. 23.4%  • Cardiac Disease:  IHD: 6% vs. 3.8%  CHF: 25.3% vs. 4.4%  • Respiratory Disease:  COPD: 17.6% vs. 9.1% | • Cardiac Complications:  35.6%4.9%  • Respiratory Complications:  26.7%7.00%  • Stroke:  0.3%0.1%  • Infection/Sepsis:  6.4%5.1% |
| ^25^Xia et al. ^2015^ | • Arrhythmias vs. Control  102 vs. 1285  •Age:  58.8 (9.1) vs. 54.0 (11.4)  •Sex (male):  66.6% vs. 63.7%  •BMI:  29.6 (6.3) vs. 27.7 (6)  •Hypertension:  38.0% vs. 32.6%  •Cardiac Disease:  IHD: 14.9% vs. 8%  •MELD Score:  31.7 (7.7) vs. 35.5 (7.1)  •History of AF:  28.4% vs. 3.6% | • Surgery Duration:  382 (165) 339 (121) |
| ^29^Manna et al. ^2013^ | • Arrhythmias vs. Control  25 vs. 279  •Age:  59.7 (7.4) vs. 50.3 (12.6)  •Sex (male):  76.00% vs. 66.3%  •BMI:  24.6 (3.6) vs. 23.8 (3.7)  •Hypertension:  96% vs. 87%  •Diabetes Mellitus:  12% vs. 4%  • Cardiac Disease:  IHD: 20% vs. 2.8%  • History of AF:  16% vs. 4.3% | • Cardiac Complications:  MI: 4% vs. 0.3%  • Infection/Sepsis:  0 vs. 0 |
| ^20^Botwinick et al. ^2011^ | • Arrhythmias vs. Control  13 vs. 236  •Age:  74 (16.43) vs. 59.66 (47.07)  •Sex (male):  38.4% vs. 47.8%  •History of AF:  23% vs. 4.6% | • Mortality:  0% vs. 1.2%  • Infection/Sepsis:  7.6% vs. 13.1% |
| ^22^Sposato et al. ^2011^ | • Arrhythmias vs. Control  7 vs. 179  •Age:  74.7 (6.9) vs. 68.3 (8.6)  •Sex (male):  100% vs. 70.95%  •Hypertension:  85.7% vs. 87.71%  •Diabetes Mellitus:  42.8% vs. 31.2%  •Cardiac Disease:  IHD: 85.7% vs. 55.8%  •Respiratory Disease:  COPD: 0 vs. 17.8% | • Surgery Duration:  153 (64) vs. 132 (28)  • Cardiac Complications:  MI: 14.2% vs. 1.1%  • Stroke:  100% vs. 1.1%  •Mortality: 0 vs. 0 |
| ^27^Sohn et al. ^2009^ | • Arrhythmias vs. Control  15 vs. 302  •Age:  74.4 (8.9) vs. 68.3 (9.5)  •Sex (male):  86.67% vs. 78.48%  •Hypertension:  60% vs. 47.6%  •Diabetes Mellitus:  13.3% vs. 20.2%  •Cardiac Disease:  IHD: 20% vs. 32.1%  CHF: 13.3% vs. 7.2%  •Respiratory Disease:  COPD: 26.6% vs. 42.7% | • Cardiac Complications:  MI 53.3% vs. 20.8% |
| ^24^Winkel et al. ^2009^ | • Arrhythmias vs. Control  30 vs. 7726  •Age:  71.9 (6.79) vs. 68.87 (5.26)  •Sex (male):  63.3% vs. 46.3%  •BMI:  22.76 (3.77) vs. 24.66 (3.48)  •Hypertension:  53.3% vs. 47.8%  •Diabetes Mellitus:  16.7% vs. 19.5% | • Infection/Sepsis:  0% vs. 0%  • Mortality  13.3%0.8% |
| ^19^Noorani et al. ^2009^ | • Arrhythmias vs. Control  20 vs. 180  •Age:  72.93 (9.13) vs. 73.16 (5.98)  •Sex (male):  90.00% vs. 87.7%  •BMI:  26 vs. 26  •Hypertension:  55.00% vs. 55.5%  •Diabetes Mellitus:  0% vs. 9.4%  •Cardiac Disease:  IHD: 10% vs. 20% VHD: 5% vs. 0.5%  •Respiratory Disease:  5% vs. 3.8%  •History of AF:  0 vs. 6.6% | • Cardiac Complications:  MI: 10% vs. 5.5%  Cardiac Failure: 25% vs. 7.7%  • Respiratory Complications:  0% vs. 0%  • Stroke:  0% vs. 0%  • Mortality:  10% vs. 3.8%  • Infection/Sepsis:  35% vs. 25.5% |
| ^28^Siu et al. ^2005^ | • Arrhythmias vs. Control  25 vs. 538  •Age:  74 (11) 67 (13)  •Sex (male):  44%43.4%  •Hypertension:  44%26.2%  •Diabetes Mellitus:  16%14.6%  •Cardiac Disease:  IHD: 20%9.8%  VHD: 0 vs. 1.4%  •Respiratory Disease:  COPD: 4% vs. 3.5% | • Surgery Duration:  176 (62) 161 (70)  • Cardiac Complications:  Cardiopulmonary: 8% vs. 4.09%  • Mortality:  8% vs. 3.1%  • Infection/Sepsis:  24.0% vs. 10.5% |

AF: Atrial Fibrillation

IHD: Ischemic Heart Disease

CHF: Congestive Heart Failure

COPD: Chronic Obstructive Pulmonary Disease

MI: Myocardial Infarction

VHD: Valvular heart Disease

# S5 – Newcastle-Ottawa scale scoring system (Quantitative study assessment)

| **Study ID** | **Type** | **Cohort Representativeness** | **Cohort Selection** | **Exposure Ascertainment** | **Demonstration that the outcome not present at start** | **Comparability** | **Outcome assessment** | **Follow-up duration** | **Follow-up adequacy** | **Total** |
| --- | --- | --- | --- | --- | --- | --- | --- | --- | --- | --- |
| ^26^Moon et al. ^2018^ | RC | * | * | * | * | ** | * | - | * | 8 |
| ^23^Leibowitz et al. ^2017^ | RC | * | * | * | * | ** | * | * | * | 9 |
| ^21^Kazaure et al. ^2015^ | RC | * | * | * | * | ** | * | - | * | 8 |
| ^25^Xia et al. ^2015^ | PC | * | * | * | * | ** | * | * | * | 9 |
| ^29^Manna et al. ^2013^ | RC | * | * | * | * | ** | - | * | * | 8 |
| ^20^Botwinick et al. ^2011^ | RC | * | * | * | * | ** | - | - | * | 7 |
| ^22^Sposato et al. ^2011^ | RC | * | * | * | * | ** | * | * | * | 9 |
| ^27^Sohn et al. ^2009^ | RC | * | * | * | * | ** | * | * | * | 9 |
| ^24^Winkel et al. ^2009^ | PC | * | * | * | * | ** | * | * | * | 9 |
| ^19^Noorani et al. ^2009^ | RC | * | * | * | * | ** | * | * | * | 9 |
| ^28^Siu et al. ^2005^ | RC | * | * | * | * | ** | * | * | * | 9 |

#

# S6 – Bias assessment results of each study with the Quality in Prognostic Studies (QUIPS) tool

| **Study ID** | **Type** | **Study**  **participation** | **Study**  **attrition** | **Prognostic**  **factor**  **measurement** | **Outcome**  **measurement** | **Study**  **confounding** | **Method to identify confounders and/or prognostic factors** | **Statistical**  **analysis and**  **reporting** | **Total** |
| --- | --- | --- | --- | --- | --- | --- | --- | --- | --- |
| ^26^Moon et al. ^2018^ | RC | Low | Moderate | Low | Low | Low | Multivariate logistic regression analyses were used to determine prognostic  factors | Low | 8 |
| ^23^Leibowitz et al. ^2017^ | RC | Low | Low | Low | Low | Low | Prognostic factors noted  to be significant on univariate analyses were evaluated in a multivariate model using stepwise  logistic regression analysis | Low | 9 |
| ^21^Kazaure et al. ^2015^ | RC | Low | Moderate | Low | Low | Low | Separate multivariate logistic regression models were  constructed to identify the risk factors and outcomes | Low | 8 |
| ^25^Xia et al. ^2015^ | PC | Low | Low | Low | Low | Low | Multivariate analyses were used to evaluate the risk factors | Low | 9 |
| ^29^Manna et al. ^2013^ | RC | Low | Low | Low | Moderate | Low | Multivariate analyses were used to evaluate the risk factors | Low | 8 |
| ^20^Botwinick et al. ^2011^ | RC | Low | Moderate | High | Moderate | Low | To determine the effect of confounder, the Breslow-Day test was applied to two 2x2 tables | Low | 7 |
| ^22^Sposato et al. ^2011^ | RC | Low | Low | Low | Low | Low | Multivariate analyses were used to evaluate the outcomes | Low | 9 |
| ^27^Sohn et al. ^2009^ | RC | Low | Low | Low | Low | Low | Multivariate analyses were used to evaluate the risk factors | Low | 9 |
| ^24^Winkel et al. ^2009^ | PC | Low | Low | Low | Low | Low | Multivariate analyses were used to evaluate the risk factors | Low | 9 |
| ^19^Noorani et al. ^2009^ | RC | Low | Low | Low | Low | Low | Multivariate regression models were  constructed to identify the risk factors | Low | 9 |
| ^28^Siu et al. ^2005^ | RC | Low | Low | Low | Low | Low | Multivariate regression models were  Used to identify the risk factors | Low | 9 |

| Study, Year, Reference | Orthopaedics (number/total) | Vascular  (number/total) | Abdominal/General (number/total) | Head and Neck (number/total) | Transplant (number /total) |
| --- | --- | --- | --- | --- | --- |
| ^26^Moon et al. ^2018^ |  |  |  |  | A: 13/13  C: 1046/1046 |
| ^23^Leibowitz et al. ^2017^ | A: 15/15  C: 395/395 |  |  |  |  |
| ^21^Kazaure et al. ^2015^ |  |  | A: 2679/2679  C: 106407/106407 |  |  |
| ^25^Xia et al. ^2015^ |  |  |  |  | A:102/102  C: 1285/1285 |
| ^29^Manna et al. ^2013^ |  |  |  |  | A: 25/25  C: 279/279 |
| ^20^Botwinick et al. ^2011^ |  |  | A: 13/13  C:236/236 |  |  |
| ^22^Sposato et al. ^2011^ |  |  |  | A: 7/7  C: 179/179 |  |
| ^27^Sohn et al. ^2009^ | - | - | - | - | - |
| ^24^Winkel et al. ^2009^ |  | A: 15/15  C:302/302 |  |  |  |
| ^19^Noorani et al. ^2009^ |  | A: 20/20  C: 180/180 |  |  |  |
| ^28^Siu et al. ^2005^ |  |  | A: 25/25  C: 538/538 |  |  |

# S7 – Distribution of the type of surgical procedures for the arrhythmia and control groups

| Surgery Type | Arrhythmia (n = 2927) | Control (n=110,885) |
| --- | --- | --- |
| Orthopedics | 0.51% | 0.35% |
| Vascular | 1.20% | 0.43% |
| Abdominal/General | 93.23% | 96.69% |
| Head & Neck | 0.24% | 0.16% |
| Transplant | 4.80% | 2.35% |
